# A transparent four-feature speech model for depression screening applicable across clinical and community settings, including assisted-living environments

**DOI:** 10.3389/fdgth.2025.1675103

**Published:** 2026-01-08

**Authors:** Kevin Mekulu, Faisal Aqlan, Hui Yang

**Affiliations:** 1 Complex Systems Monitoring, Modeling and Control Laboratory, Pennsylvania State University, University Park, PA, United States; 2 Center for Human Systems Engineering, University of Louisville, Louisville, KY, United States

**Keywords:** assisted-living, depression, digital health, interpretable AI, linguistic biomarkers, logistic regression, semantic embedding, speech analysis

## Abstract

Depression in older adults, often underrecognized and frequently conflated with cognitive symptoms, remains a major challenge in settings such as assisted-living communities. However, the need for scalable, speech-based screening tools extends across diverse populations and is not restricted to older adults or residential care. Depression in older adults is both common and frequently underdiagnosed, and while assisted-living environments represent a high-need deployment context, the present model is population-agnostic and can be validated across multiple real-world settings. Depression often co-occurs with mild cognitive impairment, creating a complex and vulnerable clinical landscape. Despite this urgency, scalable, interpretable, and easy-to-administer tools for early screening remain scarce. In this study, we introduce a transparent and lightweight AI-driven screening model that uses only four linguistic features extracted from brief conversational speech to detect depression with high sensitivity. Trained on the DAIC-WOZ dataset and optimized for deployment in resource-constrained settings, our model achieved moderate discriminative performance (AUC = 0.760) with a clinically calibrated sensitivity of 92%. Beyond raw accuracy, the model offers insights into how affective language, syntactic complexity, and latent semantic content relate to psychological states. Notably, one semantic feature derived from transformer embeddings, emb_1, appears to capture deeper emotional or cognitive tension not directly expressed through lexical negativity. Although the dataset does not contain explicit cognitive-status labels, these findings motivate future research to test whether similar semantic patterns may overlap with linguistic indicators of cognitive-affective strain observed in prior work. Our approach outperforms many more complex models in the literature, yet remains simple enough for real-time, on-device use, marking a step forward in making mental health AI both interpretable and clinically actionable. The resulting framework is population-agnostic and can be validated in assisted-living environments as one of several high-need deployment settings.

## 1 Introduction

It often begins in quiet ways: a softening of tone, a hesitation mid-sentence, a drift toward vagueness or avoidance. Depression in older adults is among the most underrecognized challenges in clinical care, particularly within assisted-living communities, where mental health resources are often limited, and symptoms may be mistaken for normal aging. What makes the situation more urgent is that depression rarely occurs in isolation. Mild cognitive impairment (MCI) frequently co-occurs with depressive symptoms in this population, creating a dual burden that increases the risk of progression to dementia, functional decline, and hospitalization [1, 2].

While depression and MCI frequently co-occur in aging populations, the present study focuses exclusively on depression detection. References to cognitive decline are provided for contextual motivation rather than as tested outcomes. This framing reflects our hypothesis that certain linguistic markers of depression may overlap with cognitive-affective patterns observed in early decline, a relationship that future longitudinal validation studies can further examine.

Despite this high-stakes context, routine depression screening in eldercare remains inconsistently implemented. Traditional tools like the PHQ-9 or Geriatric Depression Scale [3, 4] require dedicated time, trained personnel, and patient literacy, and even then, they may miss early or masked presentations of mood disturbance. Furthermore, most screening instruments offer limited visibility into the cognitive underpinnings of emotional change, overlooking subtle linguistic cues that may reflect both affect and executive strain. These limitations of traditional screening approaches in aging populations have been documented in prior work [1].

Recent advances in artificial intelligence (AI) and natural language processing (NLP) offer a promising way forward. Recent comprehensive reviews and multimodal studies have further demonstrated the role of language-based and speech-based AI in depression detection [5]. By analyzing not only what individuals say, but how they say it, we can extract early markers of both mood and cognition [6–8]. Yet many state-of-the-art AI models, particularly deep learning architectures come with a trade-off: high performance at the expense of interpretability. In clinical settings, where stakes are high and trust is critical, such opacity can hinder adoption and raise ethical concerns [9, 10].

In this study, our objective was to evaluate whether a minimal and fully interpretable set of linguistic features could reliably detect depression from spontaneous speech. Rather than optimizing for architectural complexity, we aimed to identify transparent, clinically meaningful markers that could support real-world screening workflows across diverse populations. Our focus on interpretability reflects the need for models that clinicians can easily understand, trust, and integrate into existing care pathways.

Trained on the DAIC-WOZ dataset [11–13], our model uses three handcrafted linguistic features and one derived semantic embedding. Each is interpretable, lightweight, and grounded in either psycholinguistic theory or observed speech dynamics. Most notably, a single reduced embedding dimension “*emb_1*,” emerged as a strong predictor. Through qualitative analysis, we found that this dimension may capture latent psychological conflict or cognitive-affective strain, even in the absence of overtly negative language. This raises the possibility of a new class of semantic biomarkers for affective and cognitive health, a hypothesis we believe warrants further study.

In the sections that follow, we describe our modeling approach, evaluate performance against peer systems, and explore how this transparent, text-only pipeline could support timely, scalable, and clinically meaningful screening for depression in aging populations.

Our selection of affective, syntactic, and semantic features is grounded in established psycholinguistic theory and prior evidence linking mood disorders to disrupted sentiment expression, reduced syntactic complexity, and changes in semantic coherence [14–16].

## 2 Materials and methods

### 2.1 Overview

**Figure 1:**
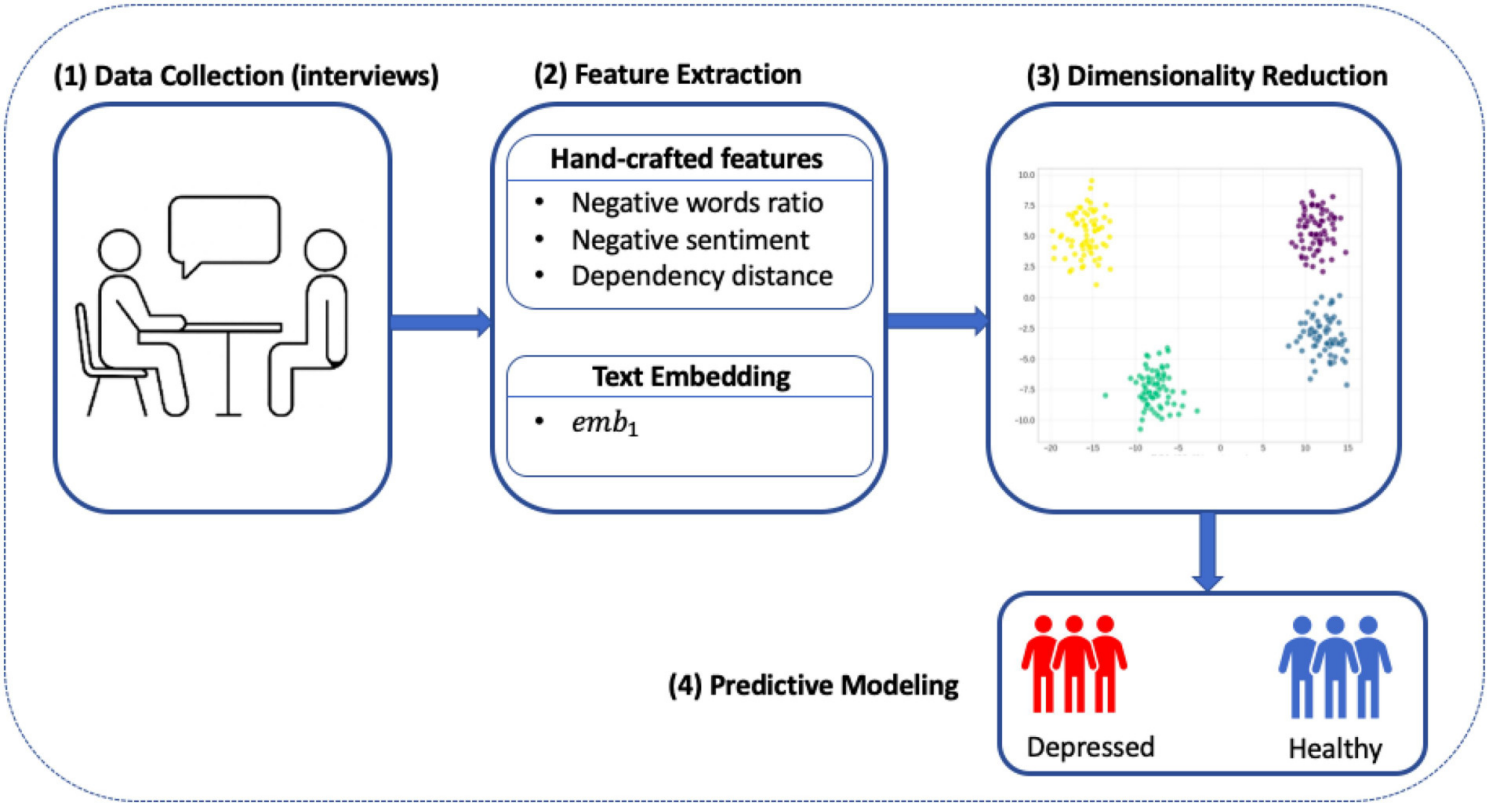
Workflow diagram for our interpretable depression screening model. Participant interviews serve as the input, followed by parallel extraction of handcrafted linguistic features and text embeddings. Embedding vectors are reduced to a single dimension, and all features are passed to a sparse logistic regression classifier.

Our aim from the outset was to design a screening pipeline that could operate not just in theory, but in the messy, time-constrained, and resource-limited context of real-world elder care. That meant simplifying everything from data inputs to model architecture, while still preserving the scientific rigor needed to make this a clinically trustworthy tool. Figure 1 summarizes our streamlined, four-stage workflow: from spoken interview to depression risk flag, all within a transparent and interpretable framework.

### 2.2 Data source and preprocessing

We used the Distress Analysis Interview Corpus—Wizard of Oz (DAIC-WOZ) dataset [11, 12], a benchmark corpus released as part of the AVEC 2017 Challenge [13]. The dataset includes transcribed semi-structured clinical interviews between participants and a virtual interviewer, with depression severity labeled based on PHQ-8 scores [3]. Each participant completed the interview in a consistent format, and binary depression labels were derived using a cutoff on PHQ-8 total scores.

To ensure that our model would generalize to real-world clinical settings, and not simply learn the dialogue structure or cues from the interviewer, we filtered out all non-participant speech. The remaining transcripts were normalized: lowercased, stripped of punctuation, and cleaned of formatting inconsistencies. After filtering, we retained 188 usable samples: 106 for training, 35 for development, and 47 for held out testing.

### 2.3 Feature extraction

We focused on capturing three types of signals: affective language, sentence complexity, and latent semantic content.

#### 2.3.1 Handcrafted features

Inspired by clinical language markers, we designed three interpretable metrics:

**Negative word ratio (neg_words_r)**—The proportion of words in a transcript that match a curated depression lexicon [17] (e.g., “hopeless,” “afraid,” “useless”).
**Negative sentiment (sent_neg)**—A scalar value computed using the VADER sentiment analysis tool, capturing the overall negative valence of the text.
**Average dependency distance (avg_dep_dist)**—A structural feature based on syntactic parsing, reflecting the average distance between tokens and their grammatical heads.

#### 2.3.2 Text embeddings

Beyond surface language and structure, we aimed to capture semantic nuance, how individuals convey affect, uncertainty, or internal conflict even without overtly depressive language. To this end, we encoded each transcript using the “all-MiniLM-L6-v2” sentence-transformer, a distilled transformer model trained via contrastive learning to produce semantically meaningful sentence embeddings [18]. Despite its lightweight architecture (22 million parameters), MiniLM retains strong representational power across a wide range of natural language processing (NLP) tasks [19], while remaining computationally efficient and well-suited for on-device inference.

### 2.4 Model training and hyperparameter optimization

We trained a sparse logistic regression model with an $\ell_{1}$ penalty to promote feature selection and interpretability. To optimize the inverse regularization parameter C, we performed a grid search across a logarithmic range of values using five-fold cross-validation on the training set. Specifically, the optimal value $C^{*}$ was selected by maximizing the average Area Under the ROC Curve (AUC) across folds:

$$C^{*}=\arg\max_{C}\frac{1}{K}\sum_{k=1}^{K}{\text{AUC}}_{k}(C)$$

where K=5 denotes the number of folds. This procedure ensured robustness to overfitting and promoted generalization to unseen data.

The resulting model retained exactly four nonzero features, each with clear psycholinguistic or semantic interpretability. To further support clinical deployment, we calibrated the model’s output probabilities using Platt scaling. A decision threshold was then chosen based on development set performance to prioritize high sensitivity (≥0.92), reflecting the design goal of minimizing false negatives in a high-risk population.

### 2.5 Evaluation strategy

Consistent with our clinical priorities, we focused on three primary metrics for model evaluation:

**Area under the curve (AUC)**—to assess the model’s ability to discriminate between depressed and non-depressed participants across thresholds.
**Sensitivity (Recall)**—to measure the proportion of truly depressed participants that were correctly flagged by the model.
**Specificity**—to understand the model’s ability to avoid false positives.

These metrics were computed exclusively on the held-out test set (*n* = 47) using the fixed high-sensitivity threshold selected during development.

## 3 Results

**Figure 2:**
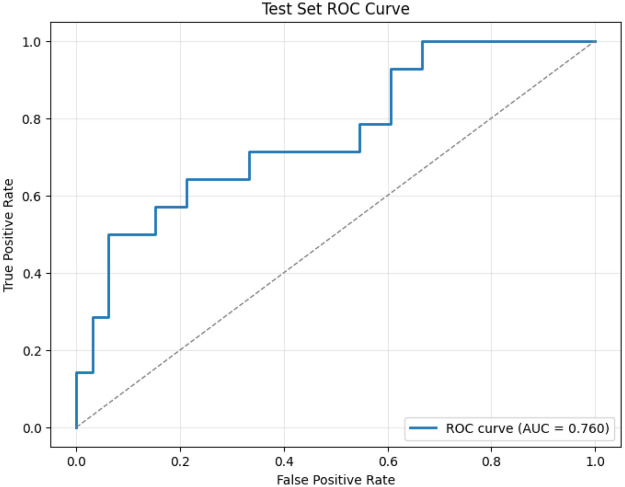
Test set ROC curve for our four-feature depression screening model. The model achieves an AUC of 0.760 on held-out data, with a clinically calibrated threshold yielding a sensitivity of 92%. This operating point reflects our design priority: maximizing true positive detection in high-risk settings like assisted living, where missed cases of depression can have serious downstream consequences. Despite its simplicity, the model maintains strong discriminative power, comparable to more complex systems while offering full interpretability and real-world deployability.

The final model was trained with C=0.6310, as determined through five-fold cross-validation on the training set. After calibration for high sensitivity, the model achieved an AUC of 0.760 on the held-out test set. At a fixed decision threshold of 0.170, selected on the development set to ensure sensitivity ≥0.92. The model reached 92% sensitivity and 57% specificity. This operating point reflects our design priority: maximizing true positive detection in high-risk settings such as assisted living. Figure 2 presents the ROC curve on the test set, demonstrating consistent discriminative performance

### 3.1 Interpreting the model: what the features tell us

Each of the four retained features carries distinct weight in shaping the model’s predictions, and each reflects a different layer of how depression can manifest in spontaneous language. The final model coefficients are summarized in Table 1, with each feature offering a distinct psycholinguistic or semantic signal.

**Table 1:** Final model coefficients for retained features.

Feature	Coefficient	Absolute value
Negative word ratio (neg_words_r)	0.458	0.458
Semantic embedding (emb_1)	0.295	0.295
Negative sentiment (sent_neg)	0.275	0.275
Dependency distance (avg_dep_dist)	−0.124	0.124

Coefficients in Table 1 represent each feature’s contribution to the model’s log-odds of predicting depression. Positive coefficients (e.g., *neg_words_r*, *emb_1*, *sent_neg*) increase the likelihood of a positive depression flag, whereas the small negative coefficient for *avg_dep_dist* indicates that syntactic simplification such as shorter dependency distances, corresponds to higher depression risk. Among these, *neg_words_r* carried the largest absolute weight, followed by the semantic dimension *emb_1*, which captures latent cognitive-affective tension not directly reflected by lexical sentiment. The coefficients were learned via an L1-regularized logistic regression, so only features with meaningful predictive value survive penalization, enhancing both sparsity and interpretability. This design allows clinicians and researchers to trace predictions back to transparent linguistic cues rather than opaque embedding vectors.

The two affective features, neg_words_r and sent_neg, provide a lexical and sentiment-based signal, respectively, both of which are well-established in prior work on depression detection. Participants who used more negative-valence language, or who expressed sadness or hopelessness explicitly, were more likely to be flagged by the model.

But the third feature, avg_dep_dist, opens a different window. This metric captures the syntactic complexity of participants’ speech: how far apart words are, on average, from the other words that govern them grammatically. Greater distances tend to reflect more nested, elaborate sentence structures, while shorter distances suggest simpler, flatter syntax. In the context of geriatric mental health, syntactic simplification has been observed in both depressive disorders and early cognitive decline [16, 20]. Thus, we interpret this feature not just as a linguistic proxy, but as a possible marker of reduced working memory or executive function. While speculative, it raises the possibility that even in speech ostensibly about mood, subtle signs of cognitive strain are already embedded.

### 3.2 Unpacking emb_1: latent meaning in speech

The most intriguing signal, however, came from emb_1, a single semantic dimension extracted via truncated Singular Value Decomposition (SVD) from a 384-dimensional transformer-based embedding. Each transcript $T_{i}$ was encoded using a pre-trained sentence-transformer model to produce an embedding vector $e_{i}\in R^{384}$. We then applied SVD across the training set:

$$E=U\Sigma V^{⊤}$$

where $E\in R^{n\times 384}$ is the embedding matrix for all n transcripts, and the columns of V represent orthogonal semantic directions. We define:

$$emb\_1(T_{i})=e_{i}\cdot v_{2}$$

where $v_{2}$ is the second right singular vector (i.e., the second column of V). This projection captures a latent semantic axis that, through exploratory correlation analysis, was found to be more predictive of depression status than the first principal component.

Unlike the handcrafted features, this signal is data-driven and abstract; not tied to any single word or syntactic structure. Yet it proved nearly as influential as the lexicon-based features.

To understand why, we manually examined transcripts with the highest and lowest values of emb_1. High-scoring interviews often included affectively loaded reflections, references to uncertainty, or socially fraught topics such as systemic injustice or personal distress. For example:

**TRANSCRIPT 426 (high emb_1)**: *“…the weather, the women, the opportunities—that’s about it. Racism, police brutality, injustice, that’s it…”*

**TRANSCRIPT 320 (high emb_1)**: *“…yes, I’m a little nervous…what do I do now? I don’t know right now…”*

By contrast, low emb_1 scores were associated with routine, fact-based, emotionally neutral language:

**TRANSCRIPT 480 (low emb_1)**: *“…New York City…once a year…both urban…east coast has a little bit maybe a little more culture…”*

**TRANSCRIPT 450 (low emb_1)**: *“…born and raised…I love the ocean…I love the hiking…”*

What emb_1 appears to capture, then, is a kind of semantic gravity, a dimension of language that reflects latent psychological weight even when overtly “depressive” words are absent.

## 4 Discussion

In assisted-living communities, where cognitive and emotional vulnerabilities often converge, the early detection of depression is more than a screening challenge; it’s a clinical imperative. Depression not only worsens functional outcomes in aging populations, but also frequently coexists with conditions like MCI, obscuring the clinical picture and delaying intervention [2, 21]. Our goal was to create a screening tool tailored for this environment, one that is interpretable, low-burden, and built to earn trust at the point of care.

This study addressed the need for an interpretable depression screening tool by developing a four-feature model derived from short speech transcripts. Unlike acoustic pipelines that require specialized hardware or noise-free conditions, this approach operates entirely on text, improving deployability in real-world care environments.

What we found was both validating and unexpected. Our sparse, four-feature model delivered strong performance (AUC = 0.760) using only a brief segment of participant speech, without relying on acoustic signals or deep black-box architectures. But beyond its numbers, the model offers insights into how depression subtly shapes language, and how those patterns can be captured and interpreted in ways that are clinically meaningful.

One such insight emerged from the inclusion of syntactic complexity, measured via average dependency distance. While simple in design, this feature draws from well-established psycholinguistic theory: reductions in grammatical complexity and clause nesting have been linked to both depression and early cognitive decline [16, 20]. These findings align with broader research linking linguistic features to cognitive and affective disorders [22]. In our model, flatter syntactic structures were associated with higher depression risk, a finding that, while not diagnostic on its own, may reflect diminished working memory or reduced executive function during speech planning.

**Figure 3:**
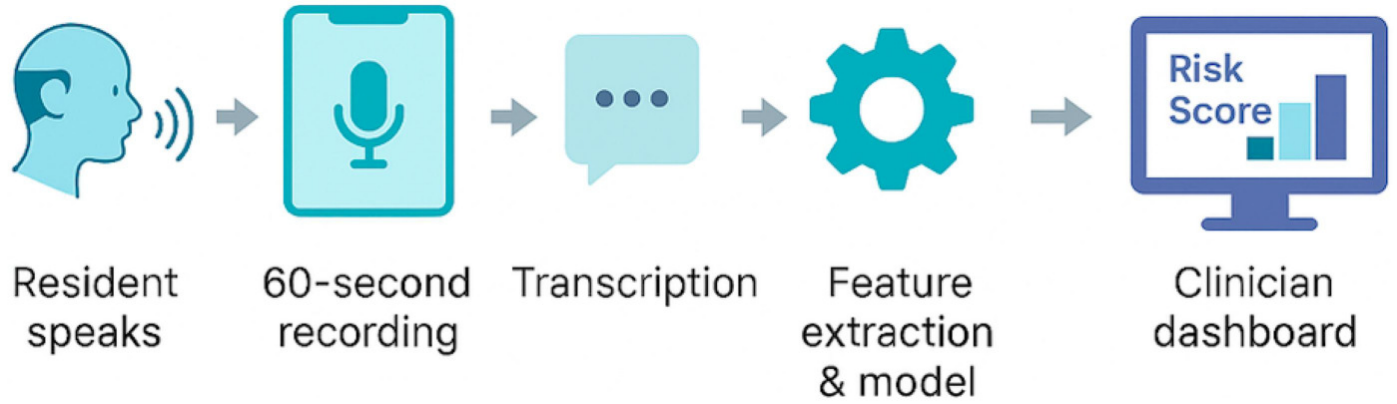
Real-world deployment pipeline for voice-based depression screening in assisted-living communities. A 60 s speech sample is recorded on a standard device, transcribed, and processed through a four-feature logistic regression model. The resulting risk score and feature explanations are displayed on a clinician dashboard for immediate review and follow-up.

Perhaps the most surprising result came from emb_1, a semantic dimension derived from a sentence-transformer embedding. Despite being abstract and data-driven, this component consistently aligned with expressions of unease, self-doubt, or affective weight, even when overtly “negative” language was absent. This opens a provocative hypothesis: that semantically derived features like emb_1 may serve as early indicators of latent cognitive-affective strain. If validated in future longitudinal work, this could mark the beginning of a new class of digital biomarkers, one capable of detecting risk before traditional screeners do. Importantly, the strength of this approach lies not only in its interpretability, but also in its readiness for real-world use. As shown in Figure 3, the full deployment pipeline, from speech capture to risk flag is lightweight, modular, and compatible with existing care workflows. This positions the tool not just as a research model, but as a scalable solution for routine screening in assisted-living communities.

## 5 Limitations and future directions

While our results are promising, several limitations must be acknowledged. First, the model was trained and evaluated on the DAIC-WOZ corpus, a structured research dataset with a standardized interview protocol. Although widely used in depression detection research, this setting may not fully reflect the linguistic variability, cultural diversity, or conversational context found in real-world assisted-living environments. Future validation on naturalistic data from clinical or residential care settings is essential.

The present study was trained exclusively on the DAIC–WOZ corpus, which contains depression assessments (PHQ-8) but no cognitive-status labels. Accordingly, any references to mild cognitive impairment (MCI) or dementia in this manuscript should be understood as conceptual motivation rather than empirical evidence. Future work integrating cognitive assessments or longitudinal data will be necessary to test whether the linguistic features identified here also correspond to early cognitive decline.

Second, our model currently relies solely on text-based features, omitting acoustic, visual, and interactional cues that could further enrich screening accuracy. While this choice was intentional, favoring deployability and robustness to noise, it also means we may be missing signals captured by multimodal systems. Additionally, the DAIC–WOZ dataset does not include participant age information. This omission limits the ability to analyze how age-related variation in language may influence depression-related speech markers. Because age is highly relevant in assisted-living populations, affecting both linguistic complexity and affective expression, future datasets should incorporate age metadata to calibrate thresholds and improve ecological validity.

Third, the semantic embedding feature (emb_1) offers strong predictive value but remains abstract. Although our qualitative analysis suggests it captures cognitive-affective tension, its exact linguistic and neuropsychological underpinnings remain speculative. Future work should explore whether such embeddings correlate with clinical assessments, neuroimaging findings, or longitudinal outcomes, particularly in individuals at risk for both depression and cognitive decline. Recent work has also highlighted opportunities and risks of applying large language models for mental-health analysis.

Finally, while our model was optimized for high sensitivity, this choice comes with a trade-off: reduced specificity. In practice, this may lead to some false positives. However, given the relatively low cost of follow-up evaluation in most assisted-living contexts, and the high cost of missed cases, we believe this trade-off is clinically justified.

Future work will focus on validating this model in real-world clinical and community deployments, integrating it with structured cognitive tasks such as verbal fluency and picture description, and evaluating its utility for longitudinal screening. An important direction is to assess whether shifts in the semantic component emb_1 may serve as early indicators of emerging depressive episodes or cognitive-affective strain. In addition, we plan to examine multilingual extensions of the system and evaluate fairness across demographic factors such as gender, race, and educational background to ensure equitable performance in diverse populations.

## 6 Conclusions

This study demonstrates that depression can be effectively screened using only a short transcript and a transparent, four-feature model, providing a generalizable framework that can be validated across diverse populations, including high-need settings such as assisted-living environments. By grounding each feature in linguistic theory and clinical relevance, we offer not just a tool, but a framework, one that balances interpretability with performance and is built for real-world use. Most notably, our analysis of a single semantic embedding dimension, emb_1, suggests the potential for new digital biomarkers that reflect latent emotional or cognitive tension even when traditional signals are absent. As aging populations grow and mental health needs in assisted-living communities intensify, tools like this, low-burden, explainable, and clinically aligned, will be essential. Future work will focus on validating this model in naturalistic settings, integrating it with structured cognitive tasks, and testing whether its semantic markers anticipate broader cognitive changes. The opportunity is clear: by listening closely to how people speak, we may detect distress long before it’s visible on a checklist.

## Data Availability

Publicly available datasets were analyzed in this study. This data can be found here: https://dcapswoz.ict.usc.edu/.
